# Investigating the Hand Ownership Illusion With Two Views Merged in

**DOI:** 10.3389/frobt.2020.00049

**Published:** 2020-04-15

**Authors:** Keisuke Okumura, Hiroki Ora, Yoshihiro Miyake

**Affiliations:** Department of Computer Science, School of Computing, Tokyo Institute of Technology, Tokyo, Japan

**Keywords:** body ownership, virtual reality, rubber hand illusion, multimodal integration, relative visibility

## Abstract

Researchers investigating virtual/augmented reality have shown humans' marked adaptability, especially regarding our sense of body ownership; their cumulative findings have expanded the concept of what it means to have a body. Herein, we report the hand ownership illusion during “two views merged in.” In our experiment, participants were presented two first-person perspective views of their arm overlapped, one was the live feed from a camera and the other was a playback video of the same situation, slightly shifted toward one side. The relative visibility of these two views and synchrony of tactile stimulation were manipulated. Participants' level of embodiment was evaluated using a questionnaire and proprioceptive drift. The results show that the likelihood of embodying the virtual hand is affected by the relative visibility of the two views and synchrony of the tactile events. We observed especially strong hand ownership of the virtual hand in the context of high virtual hand visibility with synchronous tactile stimulation.

## Introduction

Distinguishing between one's own body and external objects is an essential ability in daily life, especially when that body is threatened by an external object. Thus, body ownership, an individual's perceptual status of their body and feeling that it belongs to them (Gallagher, 2000; Tsakiris, 2010), plays a crucial role in daily existence and is considered a fundamental principle of bodily self-consciousness (Blanke, 2012). Over the past two decades, body ownership illusions have been submitted as evidence that body ownership can be flexibly modulated, deepening our understanding of the embodiment process (Botvinick and Cohen, 1998; Ehrsson, 2007; Lenggenhager et al., 2007; Kilteni et al., 2015).

Although the ability to perceive one's body is developed in a natural environment, experiments in artificial environments (e.g., virtual/augmented reality) have shown a strong potential for humans to adapt our sense of body ownership, despite the artificial context. For instance, the full body ownership illusion, which allows people the illusion of ownership over an artificial body (Maselli and Slater, 2013), is achieved in immersive virtual reality. The sense of body ownership can be perceived even using a mannequin smaller or larger than one's own body size (van der Hoort et al., 2011). Kilteni et al. reported that participants in an artificial environment can feel ownership of an arm twice or more their usual length (Kilteni et al., 2012). Compiling such evidence expands our concept of what it means to have a body, and aids designing future artificial environments. Since conception of artificial environments is unlimited, greater variation in situations should be investigated.

A prominent tool in the study of body ownership is the rubber hand illusion (RHI), which induces the illusionary perception of a dummy hand as being parts of one's own body (Botvinick and Cohen, 1998). In the RHI, illusory ownership of the dummy hand is evoked by synchronous tactile stimulation of the individual's hidden real hand and an aligned visible dummy hand placed in front of the person. In addition, participants misestimate the position of their real hand in relation to the dummy hand after stimulation, a phenomenon known as proprioceptive drift (Botvinick and Cohen, 1998; Tsakiris and Haggard, 2005). Illusory hand ownership is abolished or decreased when visuo-tactile stroking is asynchronous (Botvinick and Cohen, 1998). Many studies have clarified the conditions under which the RHI can be elicited, including visual stimuli detail, e.g., anatomical plausibility (Armel and Ramachandran, 2003; Ehrsson, 2004; Holle et al., 2011; Ide, 2013), spatial configuration (Lloyd, 2007; Kalckert and Ehrsson, 2014), and altering the shape or texture of the dummy hand (Haans et al., 2008; Bertamini and O'Sullivan, 2014). These studies have used both natural and artificial environments (IJsselsteijn et al., 2006; Sanchez-Vives et al., 2010; Kilteni et al., 2012; Martini et al., 2015).

However, most studies of the hand ownership illusion in an artificial environment have been carried out using a single view or perspective. Herein, we report a study of the hand ownership illusion during providing two overlapped first-person perspective video streams. The one was the live view obtained by a camera, and the other was recorded video of the same situation, slightly shifted toward one side. We call this situation as “two views merged in.” This work is expected to aid development of remote cooperative work, such as when specialist physicians assist a physician with less experience by sharing their perspective (Kawasaki et al., 2010; Kondo et al., 2011).

**Figure 1:**
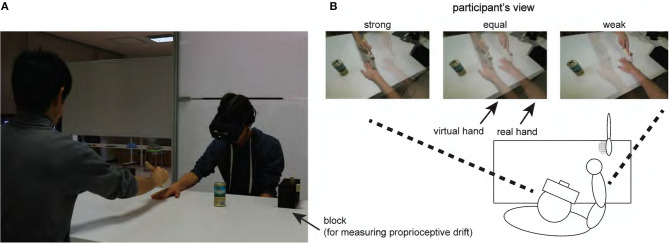
Experimental setup. **(A)** Participants wore an HMD with webcam. **(B)** During the tactile stimulation, participants were shown one of three video types (strong, equal, or weak) displaying the virtual and real hands side by side through the HMD. The hand displayed on the right hand side was their own. Each participant was instructed to move his/her head so that the empty can in each of the images overlapped completely and to maintain this head position thereafter.

In our work, these two first-person perspective views are presented by overlapped two semitransparent video streams, using weighted alpha-blending (Figure 1): one is the individual's own hand with visibility (i.e., transparency) reduced by 20% from the original; the second is another person's view of their own hand, or a dummy hand, adjusted to 80% visibility. Participants were shown these streams through a head-mounted display (HMD). The first stream is the participant's live first-person view of their real hand, with tactile events captured by the camera attached to the HMD. The second stream is a prerecorded video from a nearly identical view as that of the participant, including tactile events. The two hands (i.e., the real and virtual right hands) were aligned side by side in the overlaid video.

In this condition, we evaluated whether participants feel body ownership of the dummy hand, even though they can see and know which hand (although semitransparent) is theirs. We also tested whether the level of this embodiment could be modulated by changing the relative visibility of the embedded views. To the best of our knowledge, this is the first report on the effect on embodiment of the relative visibility of two overlapped views.

Strength of the body ownership illusion using a semitransparent body, as studied by Martini et al. (2015), decreases as the body becomes more transparent. Thus, it is natural to assume that stronger body ownership of the virtual hand will be induced by higher relative visibility of the view containing the virtual hand. Further, the RHI relies on synchronous tactile stimulation (Botvinick and Cohen, 1998). Thus, we supposed that the level of embodiment would depend on this condition; namely, asynchronous tactile stimuli would negatively affect embodiment.

To confirm this, we designed two experimental factors: (1) a tactile condition in which participants were administered paintbrush touching synchronously (*sync*) or asynchronously (*async*), and (2) a video condition. Within the *sync* and *async* conditions, we also applied three levels of relative visibility: stronger visual perception of the virtual hand with tactile events of the virtual hand (*sync-strong, async-strong*), equal visual perception of the two views (*sync-equal, async-equal*), and weaker visual perception of the virtual hand with tactile events (*sync-weak, async-weak*). Note that changing the transparency of the virtual hand also changes the transparency level of visual feedback from the participant's live first-person. Each participant experienced all six experimental conditions. Consistent with previous RHI studies (Botvinick and Cohen, 1998; Tsakiris and Haggard, 2005; Haans et al., 2008; Ide, 2013; Bertamini and O'Sullivan, 2014; Samad et al., 2015, to name just a few), we evaluated the strength of ownership over the virtual hand using a questionnaire and by measuring proprioceptive drift.

## Materials and Methods

### Participants

Thirty-one naïve, healthy adult participants with normal or corrected-to-normal vision were recruited into the study. To avoid a handedness effect, two left-handed participants (based on self-report) were later removed from analyses. Thus, we report here results from 29 right-handed participants (mean age ± standard deviation, 21.5 ± 1.9 years; 8 women, 21 men). This study was approved by the Ethics Committee of the Tokyo Institute of Technology and all methods were performed in accordance with the relevant guidelines and regulations. Written informed consent was obtained from each study participant.

### Design

The experiment was a 2 ×3 factorial design. The first variable was the touch condition, with synchronization of visuo-tactile stimulation divided into two conditions: synchronous (*sync*) and asynchronous (*async*). In the *sync* condition, the experimenter touched the participant's right hand using a paintbrush, with the touch synchronous with the stimulation of the virtual hand in the overlaid video. In contrast, in the *async* condition, stimulation of the participant's hand preceded that of the virtual view by ~0.5 s. The second variable was the video condition, or the relative visibility of the two views including visual feedback the live view. Video captures are shown in Figure 1. We blended the ratio of weighted alpha-blending in the recorded view (i.e., showing the virtual hand) with the participant's own hand in their first-person perspective. Thus, three conditions were used: stronger visual perception of the virtual hand and tactile events (*strong*, virtual hand:real hand = 8:2); equal visual perception of the two views (*equal*, virtual hand:real hand = 5:5); and weaker visual perception of the two views (*weak*, virtual hand:real hand = 2:8). These conditions were administered in random order across participants.

### Apparatus

We used an Oculus Rift (Oculus VR, LLC, Irvine, California) apparatus for the HMD and a BSW200MBK (Buffalo Inc., Nagoya, Japan) webcam. The webcam was attached to the front of the HMD using tape, to allow image capture from the first-person perspective. The webcam does not allow capture of binocular vision; however, it does support a wide angle of vision (120°). The framerate was 30 fps and we used 640 ×480 image resolution to provide the live view. The experimental program was controlled using a desktop computer, Alienware X51 (Dell Inc., Round Rock, Texas). The software was created with Unity®, a game-development platform. Weighted alpha-blending of the two images was performed using OpenCV (Bradski, 2000), an open-source library for computer vision.

#### Weighted Alpha-Blending

In the first step of generating an alpha-blended video, the program clips of one image, which is the next frame used in previous projection, from the recorded video (*virtual image*). Simultaneously, the program clips of one image from the webcam's real-time capture (*real image*). The sizes of the two images were identical. Next, the program overlays the *virtual image* onto the *real image*, according to the formula below, to produce a new image (*blended image*):

$$\begin{array}{l} blended\text{\_}image=\;\alpha \cdot virtual\text{\_}image+\;\left(1-\alpha \right)\cdot real\text{\_}image, \end{array}$$

where α represents the weight (range, 0–1). Then, a participant's projected view through the HMD is updated to the *blended image* at an updating rate of 30 fps.

### Outcome Measures

Based on previous studies (Botvinick and Cohen, 1998; Tsakiris and Haggard, 2005; Haans et al., 2008; Ide, 2013; Bertamini and O'Sullivan, 2014; Samad et al., 2015, to name just a few), we used two outcome measures: a questionnaire and proprioceptive drift.

#### Questionnaire

To quantify the perceptual experiences associated with the illusion, we used the questionnaire in Table 1, which is based on the work of Botvinick and Cohen (1998), who used nine items describing the subjective experiences of the RHI, e.g., “I felt the rubber hand was my hand.” We modified some of the items to fit our purpose and used a Japanese translation. Further, we removed the item "It felt as if my (real) hand were turning “rubbery” from the original because the meaning was not in line with our situation. Our questionnaire consisted of nine items measuring the strength of body ownership of the virtual hand (Q1–Q3), control (Q4–Q8), and one original item (Q9, “I felt the video of the merged view, which contained the virtual hand, as if it were my own sight”). Q9 assessed participants' sense of ownership of the perspective that was not their own (i.e., an “ownership of view” rating). For this experiment, the phrase “rubber hand” was changed to “virtual hand.” Participants responded to each of these nine items using a Likert scale, where −3 = “no feeling at all” and +3 = “strongly felt.” The order of the questionnaire items was randomized for each trial.

**Table 1:** Questionnaire items.

Q1	It seemed as if I was feeling the touch of the paintbrush in the location where I saw the virtual hand touching.
Q2	It seemed as though the touch I felt was caused by the paintbrush touching the virtual hand.
Q3	I felt as if the virtual hand was my hand.
Q4	It felt as if my (real) hand was drifting toward the right (toward the virtual hand).
Q5	It seemed as if I might have more than one right hand or arm.
Q6	It seemed as if the touch I was feeling came from somewhere between my own hand and the virtual hand.
Q7	It appeared (visually) as if the virtual hand was drifting toward the right (toward my hand).
Q8	The virtual hand began to resemble my own (real) hand, in terms of shape, skin tone, freckles, or some other visual feature.
Q9	I felt the video of the merged view, which contained the virtual hand, as if it were my own sight.


#### Proprioceptive Drift

Proprioceptive drift is the difference between the originally felt place of the real hand and the felt place of the hand after tactile stimulation. This measure has been used in many studies as an objective indicator of the RHI (Botvinick and Cohen, 1998; Tsakiris and Haggard, 2005; Holle et al., 2011; Samad et al., 2015; to name just a few). In this experiment, both before and after tactile stimulation, the participants were blindfolded by projecting no image in the HMD. The experimenter then set a stick horizontally 10 cm above the desk on block supports placed on either side (Figure 1A); this was also used as a ruler. The experimenter then led the participant's left index finger to the stick. The participant was instructed to use their left index finger to point to the horizontal position of their right index finger in relation to the top of the stick. The position at which they pointed was recorded and used to quantify drift from pre- to post-stimulation. After this measurement, the participant was instructed to place their left hand under the desk and the stick was removed. The visual illustration for this procedure is available (Figure S9).

### Procedure

A 3-min recording of paintbrush stroking of the virtual hand was generated prior to the experiment. The assistant whose hand was filmed sat at the table positioned to match where the participant would be placed. The assistant's right hand was placed 15 cm to the right of the table's center, with their index finger placed on a marker. The recording was captured by attaching the camera to the center of the HMD worn by the assistant. The assistant held their head in a manner that allowed the projection of the image of their right hand onto the center of the webcam on the HMD, allowing the recorded view of the virtual hand from a first-person perspective. In the recorded video, the order of finger stroking was fixed (i.e., from the little finger to the index finger, then from the index finger to the little finger) and delivered at regular intervals. Throughout the experiment and across participants, the same virtual hand video was used, regardless of participant gender, physique, or skin texture.

Before starting six sessions, the experiment was explained to the participants, including how to actualize the situation with two views merged in. In particular, they were told that the video provided through the HMD was produced by overlaying the live feed of their first-person perspective onto the pre-recorded video of the “other person's” view. The no-motion-parallax in the view that is not the participants' own triggered by the participants' head move was also well-explained. Then, to familiarize the situation, the participants experienced the short demo of the two views merged in using the HMD, for <1 min. In this demo, the unrelated video (overlooking a room, using *equal* condition) to the experiment was used as the view of not participants' own. At this time, the participants were allowed to move their heads and could experience the no-motion-parallax situation. After the demo, the participants were explained about the video as the visual stimulation used in the following six sessions. They were told that they would see two hands through the HMD and that the hand on the right side was their own hand, and the other was another person's hand. Further, they were notified that both two hands would be given paintbrush touch.

At the beginning of each session, participants were instructed to sit in the same position where the assistant had been seated in the video. The participant's right hand was placed 30 cm to the right of center on the table. Thus, the distance on the table between the virtual hand and the real hand was 15 cm. They then put on the HMD with the webcam. Before administering the tactile stimulation, we measured the estimated hand position to record proprioceptive drift, as described above. After finishing the measurement, the participant was instructed to place their left hand under the desk until the next drift measurement.

Subsequently, the experimenter projected the video in which a live feed of the participant's right hand was overlaid onto the recording of the virtual hand, depending on the video condition. The live feed was obtained from the webcam on the HMD; thus, the live feed was from the first-person perspective. The video presented the participant's hand to the right of the virtual hand (Figure 1B). To maintain the spatial alignment between the live feed and the virtual hand overlay, participants held their head in a position that maintained an overlap between an object (i.e., an empty can) placed in the center of the table in both feeds. After this alignment operation, participants were requested to stay still, especially both the head and the arms. The experimenter administered the paintbrush touch for 3 min (Figure 1A) in each condition.

The experimenter, a well-trained assistant, stimulated the participant's fingers from wrist to fingertips using the paintbrush, as shown in Figure 1B with a frequency of around one stroke per 1.5 s. The experimenter was able to see a screen showing the video projected to the participant's HMD. Thus, the experimenter could predict the next finger stroke on the virtual hand feed and adjusted their timing to either exactly, or preceding, the stroking of the virtual hand. The experimenter's screen was placed in the blind spot of the participant's live feed to avoid affecting the illusion. The participant was instructed to focus on the virtual hand during stimulation.

After each session, proprioceptive drift was measured while the participant still wore the HMD. They then removed the HMD and answered the questionnaire, followed by a short break.

### Statistical Analysis

The number of participants recruited was based on previous RHI studies using the transparency of hand approach (Martini et al., 2015). All statistical analyses were performed using R (R Core Team, 2014), version 3.4.0. The level of probability required for statistical significance was *p* < 0.05. All reports with significant differences are results after multiple comparisons (the Bonferroni-Holm method). All displayed *p*-values are not adjusted.

#### Questionnaire

As described in the Outcome Measures section, the questionnaire items were firstly categorized into embodiment-related items (Q1–Q8) or Q9. Q9 asked about the feeling of the superimposed video (i.e., merged view), which is specific to our situation. Analyses of these groupings differed since the targets for assessing were different.

For the first category (Q1–Q8), the items were further divided into measurement of body ownership (*Illusion*; Q1–Q3) or control (*Control*; Q4–Q8), according to the original work of Botvinick and Cohen (1998). To analyze the strength of body ownership, the data were integrated within each group. Thus, the data sizes for the *Illusion* statements = 29 ×3 and for the *Control* statements = 29 ×5. We first assessed whether scores differed between the *Illusion* and *Control* statements within each condition, using the Wilcoxon rank-sum test. Next, to confirm the statistical significance of *Illusion* statements between conditions (e.g., *sync-strong* vs. *sync-weak*), we analyzed the between-conditions difference within the *Illusion* statements using the Wilcoxon signed-rank test. At this time, since the data were regarded as paired, we used the Wilcoxon signed-rank test.

For Q9, we first assessed whether there was a between-conditions score difference using the Friedman test since the data of Q9 were paired within subjects. Then, as the *post hoc* test, we determined which conditions resulted in significant differences using the Wilcoxon signed-rank test, similar to the first category.

#### Proprioceptive Drift

To identify the factors affecting drift, we first performed a two-factor repeated-measures analysis of variance (ANOVA) (2 ×3) based on the results of the main factors (i.e., touch and video condition). As a *post hoc* test to determine which pair of video conditions differed significantly, we used the Tukey–Kramer method on the three visual conditions collapsed across touch conditions.

## Results

### Questionnaire

**Figure 2:**
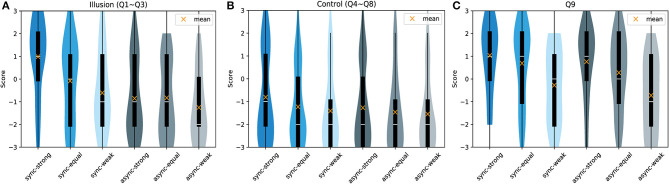
Violin plot of the questionnaire results. Participants answered the nine items shown in Table 1 for each trial. **(A)** Items measuring the strength of body ownership (*Illusion*; Q1–Q3). **(B)** Control items (*Control*; Q4–Q8). **(C)** Original item (Q9).

As stated in the Statistical Analysis section, we first divided the questionnaire items into embodiment-related questions (Q1–Q8) and our original item (Q9). The former was based on Botvinick and Cohen (1998) as measures of body ownership (*Illusion*; Q1–Q3) and control (*Control*; Q4–Q8). Violin plots of the results for each category (*Illusion, Control*, and Q9), are shown in Figure 2. The details of each item are (Figures S1–S8). We also present the illusion index (Abdulkarim and Ehrsson, 2016) (Figure S10), which was calculated as the difference between the means of the *Illusion* statements (Q1–Q3) and the *Control* statements (Q4–Q8).

Between the *Illusion* and *Control* scores, by the Wilcoxon rank-sum test, there were significant differences in the *sync-strong* condition (Z = −6.386, *p* < 0.001, *r* = 0.419), the *sync-equal* condition (Z = −4.647, *p* < 0.001, *r* = 0.305), the *sync-weak* condition (Z = −3.773, *p* < 0.001, *r* = 0.248), and the *async-equal* condition (Z = −2.816, *p* = 0.005, *r* = 0.134). In the *async-strong* (Z = −2.043, *p* = 0.041, *r* = 0.185) and *async-weak* (Z = −1.744, *p* = 0.081, *r* = 0.115) conditions, differences were no longer significant with corrections of multiple comparisons. These results suggest that, at least in all *sync* conditions, participants experienced a different feeling between the *Illusion* and *Control* items. In other words, they felt some degree of ownership of the virtual hand during synchronous touch.

We then compared the strength of body ownership of the virtual hand between the conditions by analyzing the *Illusion* statements using the Wilcoxon signed-rank test, the results of which are shown in Table 2. Scores for the *sync-strong* and *sync-equal* conditions differed significantly from those of the other conditions. Moreover, we found a significant difference between the *sync-weak* and *async-weak* conditions. None of the *async* condition pairs yielded a significant difference.

**Table 2:**
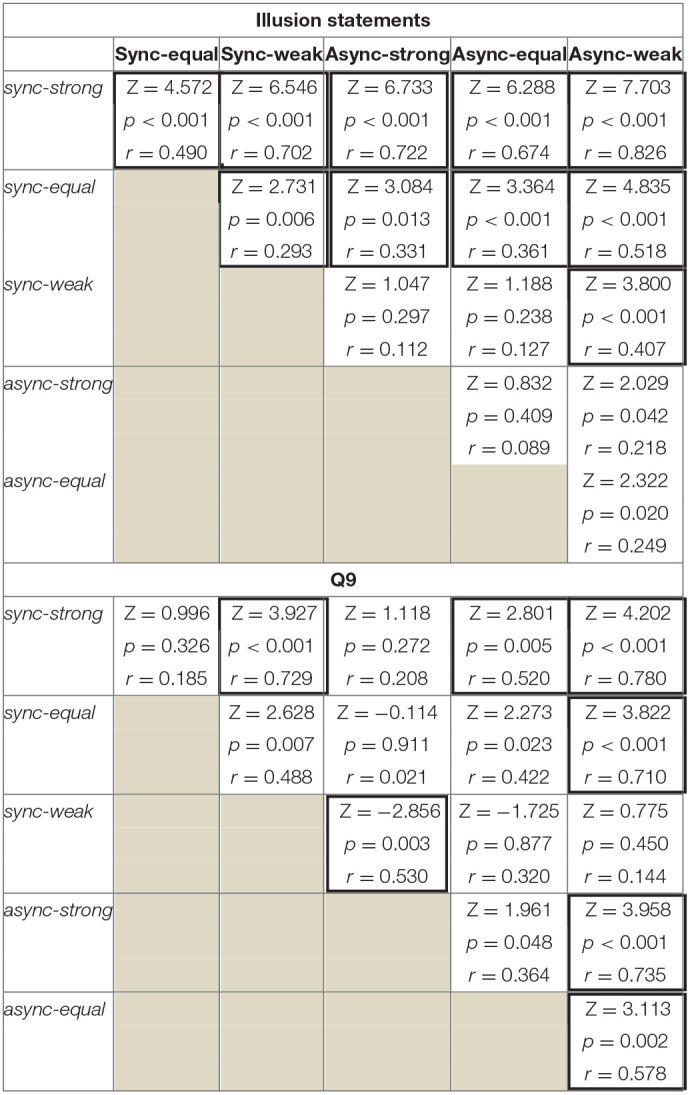
Condition pairs showing differences in the *Illusion statements* (Q1–Q3) and Q9.

*We used the Wilcoxon signed-rank test. The displayed p-values are non-adjusted. Highlighted cells with thick borders are detected as significant with multiple comparisons (using the Bonferroni-Holm method)*.

Regarding Q9, we first observed a difference between the median scores across the six conditions by the Friedman test (χ^2^(5) = 47.618, *p* < 0.001). As shown in Table 2, using the Wilcoxon signed-rank test, we then identified that the *sync-strong* condition differed significantly from the *sync-weak, async-equal*, and *async-weak* conditions. Similar to that observed for the *sync-strong* condition, *async-strong* condition scores differed from those of the *sync-weak* and *async-weak* conditions. Moreover, *sync-equal* and *async-equal* condition scores differed from that of the *async-weak* condition.

### Proprioceptive Drift

**Figure 3:**
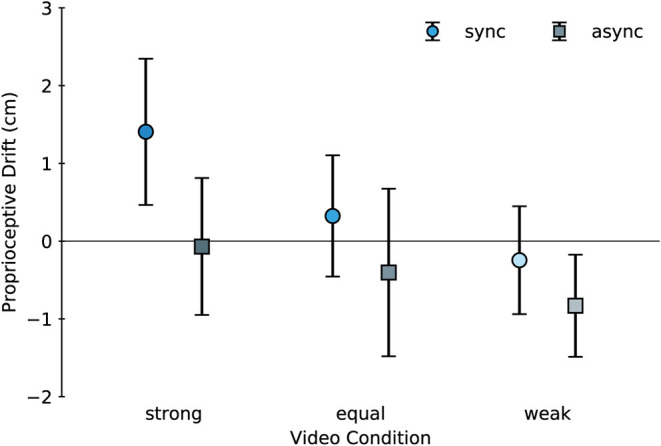
Mean proprioceptive drift. Values were positive when participants' estimations of the locations of their real hand were close to the virtual hand. The bars indicate 95% confidence intervals.

We also evaluated proprioceptive drift, which has been used as the classic index of the RHI (Botvinick and Cohen, 1998; Tsakiris and Haggard, 2005). Proprioceptive drift mean scores are shown in Figure 3. ANOVA yielded a significant main effect of both the touch condition [*F*_(1, 28)_ = 7.475, *p* = 0.011, ${\eta_{p}}^{2}$ = 0.211] and the video condition [*F*_(2, 56)_ = 4.775, *p* = 0.012, ${\eta_{p}}^{2}$ = 0.146]. No significant interaction [*F*_(2, 56)_ = 0.628, *p* = 0.538, ${\eta_{p}}^{2}$ = 0.022] was observed. Note that we confirmed that the assumption of normality of residual errors was not violated, using the Shapiro–Wilk test (*p* = 0.786). *Post hoc* analysis by the Tukey–Kramer method showed a significant difference between the *strong* and *weak* conditions (*p* = 0.011, d = 0.568); however, the remaining pairs (*strong-equal*: *p* = 0.182, d = 0.302; *equal-weak*: *p* = 0.494, d = 0.221) did not differ significantly.

## Discussion

We investigated the hand ownership illusion with two overlapped video streams. Participants could see two first-person perspective views of their arm overlapped, one is the live feed from a camera and the other a pre-recorded video of the same situation, slightly shifted toward one side. Relative visibility of these two views was modulated using a weighted alpha-blending. This also changed the transparency level of visual feedback of the live person's view. Our result supported the following three primary findings. First, from both the results of the questionnaire and proprioceptive drift, the likelihood of embodying the virtual hand was affected by the relative visibility of the virtual hand in the synchronous tactile condition. For instance, from the *Illusion* statements result, participants felt descending ownership of the virtual hand across the *sync-strong, sync-equal*, and *sync-weak* conditions. Second, according to the comparison within the *Illusion* statements, during asynchronous tactile stimulation, the relative visibility did not have a remarkable effect on subjective data asking the hand ownership compared with synchronous tactile stimuli. Third, despite the weak visual perception of the virtual hand and its corresponding tactile events, participants felt differing hand ownership between the synchronous and asynchronous touch conditions (i.e., the *sync-weak* and *async-weak* conditions, see the comparison between the *Illusion* and *Control* statements).

These findings complement those of previous and classic RHI experiments in several ways. The RHI is likely to occur when the participant's hand is fully hidden (Botvinick and Cohen, 1998) and is unlikely to occur when the real hand is fully visible (Armel and Ramachandran, 2003). Asynchronous visuo-tactile stimuli do not induce the RHI, even when visual stimuli are varied (Botvinick and Cohen, 1998; Tsakiris and Haggard, 2005). Although it is difficult to perceive the dummy hand visually (in our setting, when transparency of the virtual hand was reduced), participants are likely to feel ownership of extra objects in the synchronous visuo-tactile stimuli condition compared with the asynchronous condition (Guterstam et al., 2013; Bertamini and O'Sullivan, 2014). Our results are also consistent with the semitransparent hand ownership illusion (Martini et al., 2015), such that when transparency of one's own body is increased, body ownership is reduced.

Our questionnaire results regarding hand ownership (Q1–Q3) were affected by the video condition when tactile stimuli were synchronous, rather than by asynchronous touch. However, the tendency of the results from Q9, which aimed to assess how participants feel the superimposed video (i.e., the view that is not the participant's own), differed from that of the *Illusion* statements. One interpretation of this is that the score for Q9 was strongly affected by the video condition; conversely, the effect of the touch condition was subtle. Our interpretation of the differences in the questionnaire results observed between the *Illusion* and *Control* items in the *async-equal* condition is that a number of participants felt body ownership even during the *async* condition; these individuals may have altered the score of the *Illusion* statements (Figures S1–S3).

Our proprioceptive drift results suggest that drift was affected by both the touch and video conditions. Specifically, the relative visibility of the virtual hand seemed to be important for the drift. Based on studies that have shown a positive drift without body ownership (Holle et al., 2011; Abdulkarim and Ehrsson, 2016), it is reasonable to conclude that drift itself may be affected by the cognitive visibility of the dummy hand, i.e., drift can be affected by how visibly similar the embodiment target is to the original hand. Then, the synchronous tactile stimuli that cause body ownership tend to amplify drift, or, the asynchronous tactile stimuli have a negative effect on the drift. In the *async-weak* condition, the drift results tend to be negative due to calibration. Specifically, a negative bias may have been present across all conditions. Most participants tended to be unable to estimate the position of their right hand exactly above the desk before tactile stimulation, even though they could locate their hand precisely during practice. They tended to indicate a position slightly to the left of their real right hand. During tactile stimulation, they were able to see the correct position of their hand; therefore, we suspect that calibration of the estimated hand position was effective and produced the observed negative bias.

To interpret the induction of ownership of the virtual hand with two views merged in, in relation to findings from previous RHI studies, we mainly submit two possibilities. We changed the relative visibility of the two views and, as a consequence, changed visibility of the hands. Simultaneously, we also changed the visibility of the observed touches. Therefore, in terms of causality of ownership of the virtual hand, we can consider two possibilities; the changing the relative visibility of the two hands, or, the changing visibility of the corresponding tactile events. The first possibility can be understood in relation to the study of ownership of the semitransparent body (Martini et al., 2015). In that study, participants were shown only a virtual body, with results showing that ownership was modulated by changing the virtual body's transparency. Therefore, it is reasonable to suspect that changing relative visibility of the two hands also affected feeling of ownership. The second possibility is supported by the reported occurrence of the RHI in empty space, which is caused by presenting visuo-tactile stimuli, leading to the “invisible hand illusion” (Guterstam et al., 2013). In our study, the visual stimuli were presented such that both the virtual hand and the corresponding tactile stimuli were changed simultaneously. Conversely, in the invisible hand illusion, visibility of tactile events is clear, but the dummy hand itself is not presented, confirming the illusion. Therefore, we note that induction of ownership of the virtual hand might have been affected by the relative visibility of the corresponding tactile stimuli. Additionally, the effect of the principle of inverse effectiveness (PoIE) might have amplified the illusion. PoIE states that multisensory stimuli are most likely robustly or strongly integrated when the response elicited under the most effective unisensory condition is weak (Meredith and Stein, 1983; Holmes, 2009). The most dominant unisensory stimulation in the RHI literature is visual stimulation (i.e., visual stimulation itself is sufficient to cause the illusion) (Samad et al., 2015). In our experiments, we presented participants with a subtle virtual hand, visually. Therefore, the illusion was observed even when the participant's real hand was visible.

We also mention the supernumerary hand illusion reported by Guterstam et al. (2011). This illusion is different from the classical RHI in terms of making the real hand visible, thus participants saw two aligned right arms. Their study reported that participants had a perception that was different from that of the classical RHI, as they felt they owned a third arm. Unlike their study, our study aims to investigate the hand ownership of virtual hand with two views merged in, and we provided semi-transparent visual stimulation while adjusting the relative visibility of two views through the HMD. This makes our study original, even though the equal video condition in our setting seems to resemble their study. Additionally, the method taken here may enable to do further study of the supernumerary hand illusion by adjusting the relative visibility of two aligned hands. Note that the corresponding question to owing a third arm in our setting is Q5 (see the Supplementary Material).

Some experimental factors could affect the illusion. At first, we used the monocular camera to obtain live views of participants, however, this might contribute to downgrading the experience in the VR system. Second, in this study, we instructed participants not to move their heads to avoid that they felt a motion-parallax gap, that is, when participants move their heads, the view of not participants' own does not move. However, even though with this instruction, we could not completely suppress their heads swing a little potentially. This swing might affect the illusion. We point out that this effect is not just a spatial incongruency effect on the illusion studied so far (Lloyd, 2007; Kalckert and Ehrsson, 2014), since the incongruency here is caused by participants' move. The topic is beyond the current work, however, it is one of the interesting directions to study.

Our study made the real and the virtual arm visible at the same time, then adjusted the relative visibility of two hands (views). So far, relative visibility has been overlooked in the body ownership illusion since such a situation does not exist in a natural environment. Due to the above reason, our evidence that the relative visibility of two viewpoints affects embodiment may be valuable toward designing artificial environment interfaces. For instance, understanding this duality might help modulate the relative visibility for improving efficiency of remote-cooperative work such as (Kawasaki et al., 2010; Kondo et al., 2011; Pan et al., 2017). Further, the context of merging views may lead to interesting research questions. For example, emotions about the person with whom one is sharing a view may influence virtual embodiment. Investigation of both body ownership and self-agency will likely provide clues for how to best work in an artificial environment.

In conclusion, in our investigation of the hand ownership illusion with two views merged in, we presented participants with two overlapped first-person perspective views, while changing their relative visibility. Participants could see their arms overlapped but slightly shifted, one was from the live feed of participants' own arm and the other was from another person's pre-recorded video. Our results show that the likelihood of participants embodying the virtual hand is affected by the hands' relative visibility and synchrony of tactile events. In particular, we observed strong ownership of the virtual hand when visibility of the virtual hand was high, with synchronous tactile stimulation.

## Data Availability Statement

The datasets analyzed herein can be found in the Figshare database: https://figshare.com/s/e52c6489030dedfa2422.

## Ethics Statement

The studies involving human participants were reviewed and approved by the Ethics Committee of the Tokyo Institute of Technology. The patients/participants provided their written informed consent to participate in this study.

## Author Contributions

KO, HO, and YM designed the research. KO performed the research and analyzed the data. KO and HO wrote the manuscript.

### Conflict of Interest

The authors declare that the research was conducted in the absence of any commercial or financial relationships that could be construed as a potential conflict of interest.

## Abbreviations

RHI: rubber hand illusion
HMD: head-mounted display.
