# Supplementary material for: Investigating the Hand Ownership Illusion With Two Views Merged in
Source: Front Robot AI. 2020 Apr 15;7:49. doi: 10.3389/frobt.2020.00049 (PMC7805733; doi:10.3389/frobt.2020.00049)
Supplement: Supplementary file 1 [file Table_1.DOCX]

Supplementary Material

**Fig. S1. Q1 results.**

**Fig. S2. Q2 results.**

**Fig. S3. Q3 results.**

**Fig. S4. Q4 results.**

**Fig. S5. Q5 results.**

**Fig. S6. Q6 results.**

**Fig. S7. Q7 results.**

**Fig. S8. Q8 results.**

**Fig. S9. Illustration of measuring of proprioceptive drift.**

**Fig. S10. The illusion index, which was calculated as the difference between the means of the Illusion statements (Q1–Q3) and the Control statements (Q4–Q8).**
